# IL-1****β**** and IL-6 Upregulation in Children with H1N1 Influenza Virus Infection

**DOI:** 10.1155/2013/495848

**Published:** 2013-04-29

**Authors:** Antonio Chiaretti, Silvia Pulitanò, Giovanni Barone, Pietro Ferrara, Valerio Romano, Domenico Capozzi, Riccardo Riccardi

**Affiliations:** ^1^Department of Pediatrics, Catholic University of the Sacred Heart, A. Gemelli Hospital, Policlinico Gemelli, Largo Gemelli, 1-00168 Rome, Italy; ^2^Pediatric Intensive Care Unit, Catholic University of the Sacred Heart, A. Gemelli Hospital, 00168 Rome, Italy

## Abstract

The role of cytokines in relation to clinical manifestations, disease severity, and outcome of children with H1N1 virus infection remains thus far unclear. The aim of this study was to evaluate interleukin IL-1**β** and IL-6 plasma expressions and their association with clinical findings, disease severity, and outcome of children with H1N1 infection. We prospectively evaluated 15 children with H1N1 virus infection and 15 controls with lower respiratory tract infections (LRTI). Interleukin plasma levels were measured using immunoenzymatic assays. Significantly higher levels of IL-1**β** and IL-6 were detected in all patients with H1N1 virus infection compared to controls. It is noteworthy to mention that in H1N1 patients with more severe clinical manifestations of disease IL-1**β** and IL-6 expressions were significantly upregulated compared to H1N1 patients with mild clinical manifestations. In particular, IL-6 was significantly correlated with specific clinical findings, such as severity of respiratory compromise and fever. No correlation was found between interleukin expression and final outcome. In conclusion, H1N1 virus infection induces an early and significant upregulation of both interleukins IL1**β** and IL-6 plasma expressions. The upregulation of these cytokines is likely to play a proinflammatory role in H1N1 virus infection and may contribute to airway inflammation and bronchial hyperreactivity in these patients.

## 1. Introduction 

In the last years the world has been facing a new pandemia caused by an H1N1 influenza virus, the so-called H1N1/09 virus, which contains a unique combination of gene segments that has never been identified in humans or animals [1]. This new pandemic strain is of particular concern because of its efficient person-to-person transmission responsible for increased virulence and morbidity in humans [2, 3].

The novel influenza H1N1 virus was identified as a cause of febrile respiratory infections ranging from self-limited to severe illness both in adults and children. Recent data reported that most cases of H1N1 infection with high rate of hospitalizations occurred in children who aged 5–14 years. A small percentage of these patients can develop more complicated and severe symptoms, such as elevated fever, violent dry cough, pneumonia, and acute respiratory distress syndrome (ARDS) [4, 5], requiring admission in Pediatric Intensive Care unit (PICU) and mechanical ventilation [6]. 

Several hypotheses to explain this particular virulence of H1N1 in children were advocated, including downregulation of type 1 interferon expression, apoptosis, and hyperinduction of proinflammatory cytokines [7]. Upregulation of inflammatory cytokines, such as the TNF-a, IL-1*β*, IL-6, and IL-10, and a cytokine-mediated inflammatory response have also been documented as responsible of severity of viral lung infections [8]. Different viruses, such as respiratory syncytial virus (RSV) and adenovirus, enhanced the production of IL-6 by human macrophages influencing the susceptibility and severity of respiratory infections [9]. In addition, pulmonary and systemic inflammatory stimuli, such as hypoxia and fever, induce the biosynthesis of interleukins (ILs) in most cell types, including respiratory endothelium and mast cells [10, 11], thus determining the increase of vascular permeability and leukocyte accumulation in lung tissue [12, 13]. In the literature the inflammatory role of IL-6 and IL-1*β* in both systemic and respiratory disorders such as meningitis, head injury, and ARDS has also been reported [14, 15]. Moreover, recent studies demonstrated that influenza virus A elicits an acute inflammatory response characterized by the production of pro-inflammatory cytokines, such as IL-33 and IL-6, in infected lungs, suggesting a key role for these interleukins in the pathogenesis of respiratory epithelial cell damage and lung inflammation [16, 17]. However, the role of most cytokines in relation to clinical findings, disease severity, and outcome of children with H1N1 virus infection remains thus far unclear. Attempting to elucidate the immune mechanisms of inflammation and to clarify the role of interleukins IL-1*β* and IL-6 in children with H1N1 virus infection, we evaluated the plasma levels of these cytokines in 15 children with H1N1 infection and 15 controls with lower respiratory tract infections (LRTI), to determine whether a correlation with the expression of these molecular markers and clinical findings of these patients exists.

## 2. Patients and Methods 

### 2.1. Study Population

We conducted a prospective observational clinical study among children admitted from October 2009 to December 2010 with the diagnosis of influenza H1N1 virus infection and LRTI to the Pediatric Intensive Care Unit (PICU) and Pediatric Infectious Disease Unit (PIDU) of the “Agostino Gemelli” Hospital, Catholic University Medical School, Rome, Italy. Patients with H1N1 influenza virus infection were grouped according to age, etiology of virus infection, findings of chest radiograph, clinical and laboratory characteristics, respiratory care, and final outcome (Table 1). We also decided to differentiate the patients with H1N1 virus infection in two groups (severe and mild manifestations of H1N1 infection) based on the severity of the symptoms and on the admission to the PICU. We considered severe manifestations of H1N1 influenza infection, the presence of hypoxia at admission (SpO_2_ less than 82% in room air), ARDS requiring mechanical ventilation or noninvasive ventilation (NIV) by Helmet, oxygen supplementation by Ventimask or CPAP by face mask, severity of fever (more than 39°C at the moment of admission), presence and duration of cough, presence of specific radiological findings, such as pneumothorax (PNX), pneumopericardium, and pneumomediastinum, and other specific clinical manifestations, such as neurological involvement. Based on these admission parameters, nine patients with severe manifestations of H1N1 influenza virus infection were admitted to the PICU, while the other 6 patients with mild symptoms of H1N1 infection were admitted to the PIDU. Regarding the control group, 8 infants with severe RSV bronchiolitis were admitted to the PICU, while the other 7 children with LRTI to the PIDU. Six infants with RSV bronchiolitis admitted to the PICU underwent oxygen supplementation and NIV by Helmet, while the other 2 patients required mechanical ventilation. The other 7 infants belonging to the control group required only oxygen supplementation and symptomatic treatment (Table 2).

Oral Oseltamivir (60 mg twice daily for 5 days) was administered to all 15 patients with the diagnosis of influenza H1N1 virus infection, and supportive therapy for ARDS was started based on the severity of respiratory failure (Table 1). Fever was treated aggressively with paracetamol, while dry cough with aerosol therapy. Chest X-ray was performed within the first 6 hours of hospital admission. Eventual chest CT scan was performed in all children with H1N1 infection with particular severity of respiratory impairment or with specific findings at standard chest radiography (i.e., PNX, pneumopericardium, or pneumomediastinum). All patients were isolated at the moment of the admission based on their clinical symptoms suspected for H1N1 infection or other acute respiratory illness. The throat/nose swabs and blood samples for both laboratory studies and cytokines determination were taken at the moment of the admission. All the throat/nose swabs were sent to the microbiology for influenza virus detection and were analyzed for influenza A, B, subtypes of A by influenza real-time RT-PCR test, and RSV infection. Tables 1 and 2 reported the clinical and demographic characteristics of both patients and controls studied.

The outcome of patients was assessed upon discharge from the hospital using the Glasgow Outcome Score (GOS), which assigns a score of 1 to children who died, 2 to persistent vegetative state, 3 to severe neurologic deficits, 4 to mild neurologic deficits, and 5 to completely healthy children [18, 19].

### 2.2. Plasma Sample Collection

In H1N1 patients we collected blood samples using indwelling radial artery catheters in children admitted to the PICU or arterial puncture in children admitted to the PIDU after local painful treatment. All samples were obtained in the acute phase of the illness, at the moment of the admission of the patients, and before starting any treatment. The plasma samples were submitted for microbiological and biochemical analysis (leukocyte and platelet counts, serum C-reactive protein concentration, procalcitonin, glucose-protein concentration, electrolytes, acid-base study, BUN, etc.).

To measure interleukin levels all blood samples were centrifuged for 10 min at 5,000 rpm, and the supernatants were immediately stored at −70°C until analysis.

As controls, we used blood radial artery samples collected from children with the diagnosis of LRTI who had undergone blood sample analysis at the moment of their admission to the PICU or PIDU. 

The study was approved by the Institutional Review Board, and the parents of participating children were informed about study and provided written informed consent.

### 2.3. Interleukin Assays

IL-1*β* and IL-6 were measured from blood samples using commercial immunoenzymatic kits (Human Quantikine by R&D Systems) following the instructions of the manufacturer. The sensitivity of the assay was typically 0.70 pg/mL for IL-6 and 1 pg/mL for IL-1*β*; no cross-reactivity or interference with other related interleukins was observed. Results were represented in pg/mL, and all assays were performed in duplicate.

### 2.4. Statistical Analysis

The nonparametric Mann-Whitney test and *t*-test were used to perform statistical comparisons between children with H1N1 virus infection and LRTI control group for continuous variables. Analysis of variance was performed using Tukey-Kramer test to compare levels of IL-1*β* and IL-6 in the studied population. Linear regression analysis was used to evaluate the correlation between interleukin expression and clinical manifestations in H1N1 patients. Coefficient of determination (*R* ^2^) was taken as a measure of the goodness of fit of the model. A *P* value <0.05 was considered significant. Statistical and database software used included GraphPad version 5.0 (GraphPad Software, San Diego, CA, USA) and Microsoft Office Excel 2007 (Microsoft Corporation, Redmond, WA, USA), respectively. 

## 3. Results

### 3.1. Clinical and Laboratory Differences between H1N1 Patients and Controls

 We include in this study 15 patients with H1N1 virus infection and 15 children with LRTI. Patients with H1N1 infection aged 2.8 years to 17.3 years, with a mean age of 7.9 years, while children with LRTI aged 1.1 years to 6.3 years, with a mean age of 3.7 years. Nine children with severe H1N1 virus infection were admitted to our PICU due to the severity of their respiratory compromise, while the other 6 patients to the PIDU. Among children with LRTI, 8 out of 15 were admitted to the PICU with the diagnosis of severe RSV bronchiolitis, while the other 7 were admitted to the PIDU (4 with diagnosis of non-RSV bronchiolitis and 3 with diagnosis of influenza A (H2N3) virus infection). Regarding clinical differences between the two groups, H1N1 patients experienced higher median fever (39.2°C) compared to controls (37.7°C) (*P* < 0.0001). Cough was a common symptom in both groups. However, H1N1 patients more frequently suffered from a dry and longer cough compared to LRTI patients (median 6 days versus 4 days) (*P* < 0.0001). The most frequent pulmonary abnormalities at chest X-ray were represented by pneumonia and pulmonary consolidation in the H1N1 patients, while in LRTI children we detected atypical findings, such as hyperinflated lungs and segmental pulmonary atelectasias. Two patients with H1N1 infection showed PNX, while another three children showed severe respiratory complications, such as pneumopericardium, pneumomediastinum, and pneumorrhachis at chest CT scan (Table 1). No pulmonary or systemic complications were referred to LRTI group. No differences in clinical manifestations, such as gastrointestinal and neurological symptoms, have been reported between the groups. Regarding laboratory tests (blood cells and platelet count, serum C-reactive protein, procalcitonin, GOT, GPT, CTN, and urea) no significant differences were detected between H1N1 patients and LRTI controls. All children, both patients and controls, had a good outcome without any significant complications (GOS 5), but H1N1 patients had a significantly longer time of hospitalization compared to the control group (9 days versus 3 days: *P* = 0.0013).

### 3.2. Interleukin Expression in H1N1 Patients and Controls

**Figure 1:**
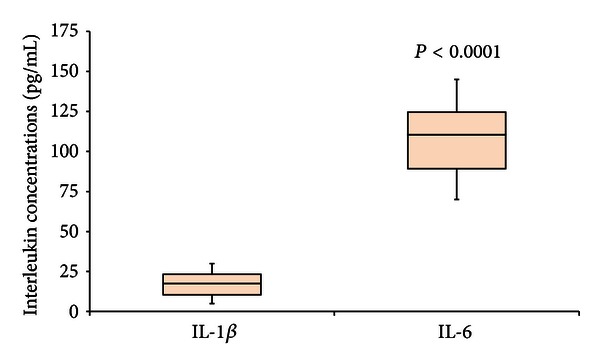
Levels of IL-1*β* and IL-6 in H1N1 patients are shown in the figure. Box plot representation was used. The level of IL-6 was significantly higher than the level of IL-1*β* (*P* < 0.0001).

**Figure 2:**
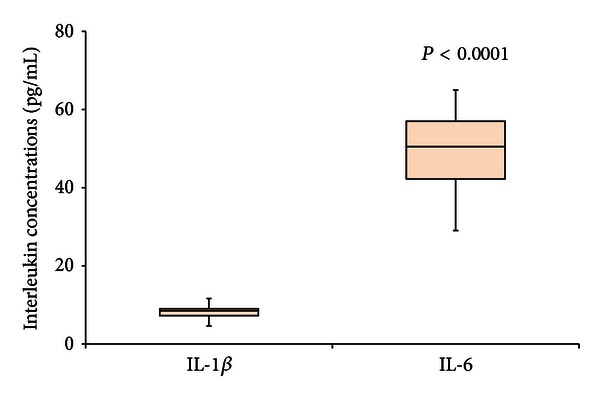
Levels of IL-1*β* and IL-6 in LRTI patients are shown in the figure. Box plot representation was used. IL-6 level was significantly higher compared to IL-1*β* (*P* < 0.0001).

 In H1N1 patients we detected different plasma levels of interleukins. In these patients we found significantly (*P* < 0.0001) higher levels of IL-6 (108.1 ± 22.8 pg/mL) compared to IL-1*β* (17.2 ± 7.9 pg/mL) (Figure 1). Also in LRTI patients the mean plasma levels of IL-6 were significantly higher compared to the levels of IL-1*β* (49.0 ± 10.3 pg/mL versus 8.1 ± 2.2 pg/mL) (*P* < 0.0001) (Figure 2).

### 3.3. Plasma Level Differences of Interleukin Expression between H1N1 Patients and Controls

**Figure 3:**
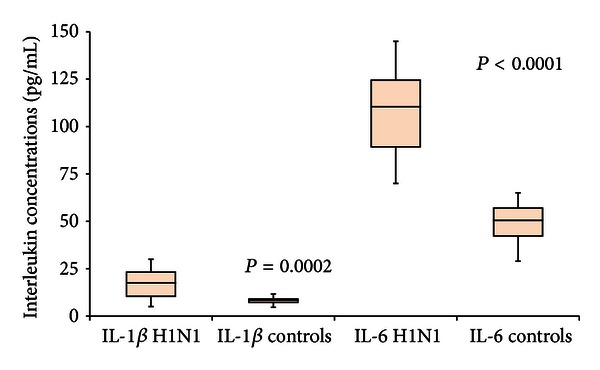
Box plot representation was used. H1N1 patients had significantly higher levels of IL-1*β* (*P* = 0.0002) and IL-6 (*P* < 0.0001) when compared to controls.

 Significantly higher levels of interleukin IL-6 and IL-1*β* were demonstrated in all patients with H1N1 infection compared to controls. Compared with LRTI patients, H1N1 patients displayed significantly increased plasma levels of IL-6 (108.1 ± 22.8 pg/mL versus 49.0 ± 10.3 pg/mL; *P* < 0.0001) and IL-1*β* (17.2 ± 7.9 pg/mL versus 8.1 ± 2.2 pg/mL; *P* < 0.0002) (Figure 3). 

### 3.4. Correlation between Interleukin Expression with Disease Severity and Clinical Manifestations in H1N1 Patients

**Figure 4:**
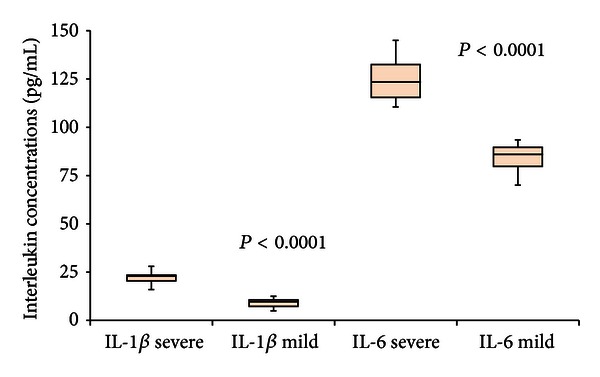
Box plot representation was used. H1N1 patients with severe disease had significantly higher levels of IL-1*β* (*P* < 0.0001) and IL-6 (*P* < 0.0001) when compared to patients with mild symptoms.

 To elucidate the association between interleukin expression and disease severity, we analyzed their plasma levels both in patients with severe (9 patients) and mild symptoms (6 patients) of H1N1 influenza virus infection. Compared to the mild patients, severe H1N1 patients produced significant higher levels of IL-1*β* (22.6 ± 4.7 pg/mL versus 9.1 ± 2.8 pg/mL; *P* < 0.0001) and IL-6 (124.1 ± 11.8 pg/mL versus 84.0 ± 8.6 pg/mL; *P* < 0.0001) (Figure 4).

**Figure 5:**
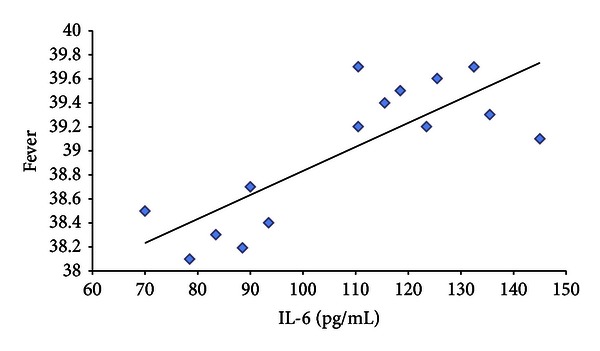
A scatter plot shows the relationship between fever and IL-6 plasma levels. The line represents the regression line (linear regression equation: fever = 0.02 × IL-6 + 36.8 *R* ^2^ = 0.64).

**Figure 6:**
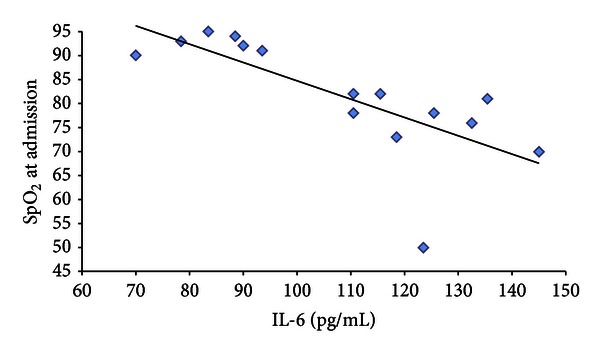
A scatter plot show the relationship between SpO_2_ at admission in room air and IL-6 plasma level. The line represents the regression line (linear regression equation: SpO_2_ = −0.38 × IL-6 + 122.9*R* ^2^ = 0.53).

 Moreover, to verify whether there was a correlation between interleukin up-regulation and clinical manifestations in H1N1 patients, we compared the plasma levels of these cytokines with some clinical symptoms referred to the patients. In particular, we detected a positive correlation between plasma level of IL-6 and fever with a coefficient of determination of 0.64 (*P* = 0.0004) (Figure 5). Finally we found a negative correlation between IL-6 plasma level and SpO_2_ at admission in room air with a coefficient of determination of 0.53 (*P* = 0020) (Figure 6). No significant correlations were reported between interleukin expression and other clinical and laboratoristic parameters, such as biochemical markers of inflammation (C-reactive protein and procalcitonin), respiratory care, systemic complications, and, finally, outcome of all children, with H1N1 virus infection.

## 4. Discussion

 Our study, despite the limited patient sample so far evaluated, provides evidence that H1N1 virus infection induces an early and significant up-regulation of interleukin IL-1*β* and IL-6 plasma levels suggesting that these cytokines are responsible for different molecular reactions leading to airway inflammation and disease severity. Compared to LRTI controls, H1N1 infected children showed a strongly higher production of both IL-1*β* and IL-6 soon after virus lung infection, and this overexpression seems to correlate with the severity of clinical compromise assessed upon admission. We also observed that in H1N1 patients with more severe clinical manifestations of disease, plasma levels of IL-1*β* and IL-6 were significantly upregulated compared to H1N1 mild patients and that this over expression was correlated with some specific clinical manifestations and a longer time of hospitalisation. More in particular, IL-6 up-regulation was significantly correlated with the severity of respiratory compromise, testified by a lower SpO_2_ at admission and higher fever observed in this subset of children, as previously reported in patients with H1N1 virus infection [7]. No differences were reported between plasma expression of these factors and final outcome of patients and controls. 

 To date it is difficult to explain the exact role of ILs in the mechanisms of virus host response, because both pro-inflammatory and immunoprotective actions have been reported in previous researches. H1N1 virus infection causes the activation of the host macrophages and lymphocytes determining the release of pro-inflammatory cytokines. The increased expression of pro-inflammatory cytokines into the lung tissue may lead to higher blood vessel permeability, phagocytic cell recruitment, apoptosis of lung epithelial cells, and release of neutrophil-derived enzymes, such as myeloperoxidase and elastase, responsible of severity of acute lung injury [20]. Our results are in agreement with these studies, as we showed a significant correlation between ILs up-regulation and severity of respiratory compromise in children with H1N1 virus infection. There are some possible explanations for this relationship. Up-regulation of IL-1*β* and IL-6 may affect lung functioning because hypoxia is in turn responsible for the endogenous cytokine production after H1N1 lung infections [10]. Cytokine up-regulation may cause epithelial cell damage through different mechanisms. ILs have a direct toxic effect by increasing the production of nitric oxide synthase, cyclooxygenase, and free radicals and by favouring the release of the excitatory amino acid in experimental model of neurotoxicity and also in patients with severe sleep apnea [21, 22],thus determining impaired pulmonary function [23]. Previous studies, in fact, reported the correlation between IL-1*β* and IL-6 up-regulation and some clinical and radiological findings, such as pneumonia and ARDS, both in experimental animal models and in children with naturally acquired seasonal influenza A [24–27]. More in particular, IL-1*β* and IL-6 have been identified as specific markers of the severity of acute lung injury during H1N1 influenza virus infection [8] and it has also been reported that IL-1*β* is an early and useful biomarker of the severity and progression of lung inflammation in patients undergoing mechanical ventilation and unresponsive to anti-microbiological treatment [28]. Our results are consistent with these previous researches because children with more severe clinical and radiological manifestations of H1N1 disease severity, such as ARDS and longer and dry cough, elicited a more intensive production of IL-1*β* and IL-6 than H1N1 mild patients, suggesting that this up-regulation exerted a key role in biochemical and molecular processes affecting the lung soon after the infection leading to the development of airway inflammation and bronchial hyperreactivity [29, 30].

 Up to now it is difficult to explain if the observed ILs up-regulation in H1N1 patients can represent a protective mechanisms for respiratory cell survival or it is secondary to a loss of physiological control of ILs biosynthesis. Available clinical and experimental data does not permit a definitive clarification of these findings. ILs plasma levels increase in several inflammatory diseases, such as allergen provocation and asthma. Recently, lymphocytes and in particular activated T cells were revealed to express ILs receptors in the experimental animal model of pulmonary sarcoidosis and chemical lung injury [31, 32]. So, it is possible that ILs upregulation is secondary to lymphocytes rapid activation by H1N1 virus infection and that this over-expression represents an important process in the mechanisms of inflammatory host response after viral lung infections [33, 34]. 

 Previous studies, in fact, reported that different viral lung infections are associated with early up-regulation of cytokine biosynthesis suggesting that the changes of ILs release may contribute to the development of airway inflammation and bronchial hyperreactivity [34]. In our study, IL-1*β* and IL-6 up-regulation, observed early after H1N1 virus infection, was consistent with the timing of cytokine expression in experimental models of virus infected human alveolar macrophages, suggesting that this over expression plays a key role in the mechanisms of inflammatory lung response [35]. The significant correlation between ILs upregulation and severity of H1N1 virus infection observed in our patients might reflect an endogenous attempt against molecular mechanisms activated in the epithelium cells of infected lung suggesting that ILs up-regulation acts in different fashion to amplify and propagate inflammation in the airways. However, given that the statistical power to find a statistically significant association in a model of 15 patients is very low, we need to be very cautious on interpreting these data, because only limited information is available on ILs expression in children with viral lung infections.

In conclusion, our observations provide new evidence that an immune response is activated at the early stage of pandemic H1N1 influenza virus infection with up-regulated production of plasma interleukins IL-1*β* and IL-6. These findings are consistent with previous experimental and clinical studies confirming a key role for both of these interleukins in the pathogenesis of airway inflammation and bronchial hyperreactivity during virus lung infections. The increased expression of these cytokines may together be the underlying cause of the observed clinical symptoms in severe H1N1 patients, and defining the relationships between ILs expression and the pathophysiology and clinical manifestations of H1N1 may help to shed light on the molecular pathogenesis of H1N1 influenza and other human viral lung infections. Further clinical and experimental investigations are necessary to identify the ILs target cells in the damaged lung and to discover possible clinical applications of ILs in children with H1N1 influenza virus and other viral lung infections.

## Figures and Tables

**Table 1:** Clinical and radiological findings, respiratory assessment, and complications of H1N1 infected children. In bold the more severe H1N1 patients.

Patients	Fever at first day of admission	Duration of cough (days)	SpO_2_ at admission in room air	Chest X-ray	Respiratory care	Antimicrobial therapy	Complications	Length of stay in hospital (days)	Outcome (GOS)
**1**	39.6 ± 0.5	**8**	**78%**	**Pneumothorax**	**O**_2_ ** supplementation by Ventimask**	**Ceftriaxone,** **Clarithromycin, and** **Oseltamivir**	**None**	**11**	**5**

**2**	39.7 ± 0.4	**6**	**78%**	**Pneumonia**	**CPAP by Helmet** **(1 day) + ** **O**_2_ **by Ventimask**	**Ceftriaxone,** **Oseltamivir**	**None**	**10**	**5**

**3**	39.1 ± 0.6	**8**	**70%**	**Interstitial pneumonia, pleural effusion**	**NIV by full-face mask, EI/MV** **(2 days ), CPAP by Helmet** **(4 days)**	**Ceftriaxone,** **Clarithromycin, and** **Oseltamivir**	**Pneumorrachis**	**19**	**5**

**4**	39.3 ± 0.6	**6**	**81%**	**Pneumonia**	**O**_2_ **supplementation by Ventimask**	**Ceftriaxone,** **Oseltamivir**	**None**	**9**	**5**

**5**	39.4 ± 0.7	**7**	**82%**	**Bilateral pulmonary infiltrates**	**O**_2_ **supplementation by Ventimask**	**Ceftriaxone,** **Oseltamivir**	**None**	**9**	**5**

**6**	39.2 ± 0.4	**7**	**50%**	**Bilateral pulmonary consolidation**	**EI/MV** **(11 days), CPAP by Helmet** **(4 days)**	**Ceftriaxone,** **Clarithromycin, and** **Oseltamivir**	**None**	**27**	**5**

**7**	39.7 ± 0.6	**6**	**76%**	**Bilateral pulmonary infiltrates**	**O**_2_ **supplementation by Ventimask**	**Ceftriaxone,** **Oseltamivir**	**Pneumomediastinum**	**11**	**5**

**8**	39.2 ± 0.3	**5**	**82%**	**Pneumonia**	**O**_2_ **supplementation by Ventimask **	**Ceftriaxone,** **Oseltamivir**	**Pneumopericardium**	**18**	**5**

**9**	39.5 ± 0.4	**9**	**73%**	**Pneumothorax,** **bilateral pulmonary consolidation **	**NIV by full-face mask**	**Ceftriaxone,** **Oseltamivir**	**None**	**18**	**5**

10	38.5 ± 0.5	7	90	Interstitial pneumonia	O_2_ supplementation	Klaritromicin, Oseltamivir	None	7	5

11	38.7 ± 0.2	4	92	Normal	O_2_ supplementation	Oseltamivir	None	4	5

12	38.2 ± 0.4	5	94	Normal	None	Oseltamivir	None	3	5

13	38.3 ± 0.7	5	95	Normal	None	Oseltamivir	None	3	5

14	38.4 ± 0.6	6	91	Hyperinflated lung	O_2_ supplementation	Oseltamivir	None	6	5

15	38.1 ± 0.4	6	93	Normal	None	Oseltamivir	None	3	5

CPAP: continuous positive airway pressure; EI: endotracheal intubation; MV: mechanical ventilation.

**Table 2:** Clinical and radiological findings, respiratory assessment, and complications of LRTI children. In bold the more severe LRTI patients.

Patients	Fever at first day of admission	Duration of cough (days)	SpO_2_ at admission in room air	Chest X-ray	Respiratory care	Antimicrobial therapy	Complications	Length of stay in hospital (days)	Outcome (GOS)
**1**	37.4 ± 0.3	**4**	**84%**	**Segmental pulmonary atelectasia**	**CPAP by Helmet** **and ** **O**_2_ ** supplementation **	**Amoxicillin**	**None**	**4**	**5**

**2**	37.5 ± 0.2	**5**	**82%**	**Segmental pulmonary atelectasia and hyperinflated lung**	**Mechanical ventilation** **and ** **O**_2_ ** supplementation**	**Amoxicillin,** **Ceftriazone**	**None**	**5**	**5**

**3**	38.1 ± 0.1	**5**	**85%**	**Hyperinflated lung**	**O**_2_ **supplementation by face mask**	**Amoxicillin**	**None**	**3**	**5**

**4**	38.3 ± 0.2	**3**	**81%**	**Hyperinflated lung**	**CPAP by Helmet** **and ** **O**_2_ ** supplementation **	**Amoxicillin**	**None**	**3**	**5**

**5**	37.4 ± 0.4	**4**	**82%**	**Segmental pulmonary atelectasia**	**CPAP by Helmet** **and ** **O**_2_ ** supplementation **	**Amoxicillin**	**None**	**2**	**5**

**6**	37.2 ± 0.6	**3**	**81%**	**Hyperinflated lung**	**CPAP by Helmet** **and ** **O**_2_ ** supplementation**	**Amoxicillin**	**None**	**3**	**5**

**7**	38.5 ± 0.6	**4**	**80%**	**Hyperinflated lung**	**Mechanical ventilation** **and ** **O**_2_ ** supplementation **	**Claritromicina** **Amoxicillin**	**None**	**4**	**5**

**8**	38.1 ± 0.3	**5**	**82%**	**Segmental pulmonary atelectasia**	**CPAP by Helmet** **and ** **O**_2_ ** supplementation **	**Amoxicillin**	**None**	**4**	**5**

9	37.5 ± 0.2	4	93%	Segmental pulmonary atelectasia	O_2_ supplementation	Amoxicillin	None	5	5

10	37.5 ± 0.3	3	91%	Interstitial pneumonia	O_2_ supplementation	Claritromicina	None	5	5

11	37.7 ± 0.5	4	88%	Normal	O_2_ supplementation	Amoxicillin	None	4	5

12	37.5 ± 0.3	3	93%	Normal	None	Amoxicillin	None	2	5

13	38.1 ± 0.4	3	91%	Normal	O_2_ supplementation	Amoxicillin	None	3	5

14	38.3 ± 0.6	3	86%	Hyperinflated lung	O_2_ supplementation	Amoxicillin	None	3	5

15	37.8 ± 0.5	4	90%	Normal	O_2_ supplementation	Amoxicillin	None	3	5

CPAP: continuous positive airway pressure; EI: endotracheal intubation; MV: mechanical ventilation.
